# Honeybee associated *Aspergillus niger* AW17 as a source of selective anticancer compounds with cytotoxicity evaluation in human cancer cell lines

**DOI:** 10.1038/s41598-025-18565-y

**Published:** 2025-09-12

**Authors:** Hamada A. Zina, Mohamed H. Kalaba, Abdelghany S. Shaban, Ahmed A. Elrefaey, Hesham M. Mahdy, Abdullah Haikal

**Affiliations:** 1 Botany and Microbiology Department, Faculty of Science, Al-Azhar University, Cairo, 11884 Egypt; 2 Department of Pharmacognosy, Faculty of Pharmacy, Mansoura University, Mansoura, 35516 Egypt

**Keywords:** *Aspergillus Niger*, Honeybee microbiome, Anticancer activity, Cell cycle arrest, Apoptosis, Confocal microscopy, Oleic acid, Pachymic acid, Ecological niche, Membrane integrity, Biochemistry, Biotechnology, Microbiology

## Abstract

Cancer remains a leading cause of death worldwide, necessitating the discovery of novel therapeutic agents with improved efficacy and selectivity. This study investigated the anticancer potential of *Aspergillus niger* strain AW17 isolated from honeybees, a previously unexplored ecological niche for anticancer drug discovery. The fungal isolate was characterized through morphological, microscopic, and molecular methods, confirming its identity as *Aspergillus niger.* Chemical profiling using GC-MS and UPLC-MS/MS revealed a complex composition including high oleic acid content (28.88%) and unique compounds like pachymic acid (18.93%), rarely associated with *A. niger.* The extract’s anticancer activity was evaluated against colorectal (Caco-2), liver (HepG2), breast (MCF-7), and lung (A549) cancer cell lines using MTT assay, confocal microscopy with AO/PI staining, cell cycle analysis, and apoptosis detection. Results demonstrated remarkable selective cytotoxicity of the Aspergillus niger extract against cancer cell lines, following the sensitivity hierarchy HepG2 (IC_50_ = 5.22 µg/ml) > Caco-2 (26.78 µg/ml) > A549 (34.18 µg/ml) > MCF-7 (55.91 µg/ml), while sparing normal cell lines significantly, with IC_50_ values of 1454.7 µg/ml for WI-38 and 668.3 µg/ml for HFB-4, indicating high cancer cell selectivity. Confocal microscopy confirmed membrane integrity loss as a primary cytotoxic mechanism. Cell cycle analysis revealed cell-type specific arrest patterns, with Caco-2 and HepG2 exhibiting G1 arrest, while MCF-7 and A549 showed S phase accumulation. Flow cytometry indicated distinct death mechanisms, with Caco-2 undergoing early apoptosis and substantial necrosis, HepG2 showing predominant late apoptosis, and A549 displaying primary necrotic death. These findings highlight the potential of honeybee-associated fungi as sources of novel anticancer compounds with remarkable selectivity, providing new avenues for targeted cancer therapy development and demonstrating the value of exploring specialized ecological niches in natural product discovery.

**Supplementary Information:**

The online version contains supplementary material available at 10.1038/s41598-025-18565-y.

## Introduction

Cancer is a leading global health challenge and the second most common cause of death, responsible for about 10 million deaths in 2020. Incidence is projected to rise to 28.4 million new cases by 2040—a 47% increase—with colorectal, liver, breast, and lung cancers accounting for over 40% of cancer-related deaths^1–3^.

Despite advances in surgery, chemotherapy, radiotherapy, and immunotherapy, cancer treatment faces persistent challenges, including lack of specificity, severe side effects, and the emergence of drug resistance, which often leads to treatment failure. Additionally, the global economic burden of cancer is substantial, estimated at $1.16 trillion annually, underscoring the urgent need for more effective, affordable, and accessible^4–6^.

This pressing global health crisis necessitates continuous exploration of novel therapeutic approaches. A particularly promising avenue involves harnessing natural products as potential anticancer agents. Throughout history, natural products have served as invaluable sources of bioactive compounds, with approximately 60% of currently approved anticancer drugs derived directly or indirectly from natural sources. The extraordinary chemical diversity found in nature, shaped by millions of years of evolution, offers unique structural scaffolds and bioactive properties that synthetic chemistry often struggles to replicate^7–9^.

Microorganisms represent a particularly rich yet underexplored reservoir of bioactive compounds with therapeutic potential. The microbial world, encompassing bacteria, fungi, and various other microorganisms, has contributed significantly to our medical armamentarium, with notable examples including penicillin, cyclosporine, and statins. Fungi, in particular, have emerged as prolific producers of secondary metabolites with diverse biological activities. The genus *Aspergillus*, with over 350 recognized species, has yielded numerous bioactive compounds, including lovastatin (a cholesterol-lowering agent) and fumagillin (an antibiotic and antitumor agent^10–12^.

Recent scientific interest has increasingly focused on microorganisms from unique ecological niches, based on the premise that unusual habitats may drive the evolution of novel metabolic pathways and bioactive compounds^13^. This ecological approach to natural product discovery has opened new frontiers in the search for anticancer agents. One such distinctive ecological niche that has gained attention is the honeybee microbiome, the complex community of microorganisms that exists within and around honeybees and their hives^14,15^.

The honeybee (*Apis mellifera*) microbiome represents a fascinating and largely untapped source of microbial diversity, comprising bacteria and fungi that influence bee health, nutrition, and colony dynamics. This specialized community is shaped by diet (nectar and pollen), environmental exposures during foraging, and hive interactions, potentially harboring microorganisms with unique metabolic capabilities adapted to this niche^16–18^.

Fungi isolated from honeybees and their hives such as *Aspergillus*,* Penicillium*,* Mucor*,* and Cladosporium* represent a promising source of bioactive compounds. Their entry into the hive via pollen, nectar, or the environment, and their diverse ecological relationships with bees, may drive the production of unique secondary metabolites adapted to this specialized niche^18–20^.

Despite the promising potential of bee-associated fungi as sources of bioactive compounds, significant knowledge gaps persist in this area. Limited research has been conducted on the systematic isolation, characterization, and bioactivity screening of fungi from honeybee sources. Moreover, the potential anticancer properties of metabolites produced by these organisms remain largely unexplored. This represents a critical gap in current natural product research, given the urgent need for novel anticancer compounds with improved efficacy and selectivity. The present study addresses these knowledge gaps by focusing on *Aspergillus niger* strain AW17, a fungal isolate obtained from honeybees. *A. niger* is a filamentous fungus known for its remarkable metabolic versatility and industrial applications in enzyme and organic acid production^21–23^. However, strains of *A. niger* from specialized ecological niches, such as the honeybee microbiome, may possess unique metabolic capabilities and produce distinctive secondary metabolites not found in their counterparts from more common environments.

The aim of this study is to explore the anticancer potential of *Aspergillus niger* strain AW17 isolated from honeybees, through chemical characterization of the extract using complementary analytical techniques (GC-MS and UPLC-MS/MS) and systematic evaluation of the extract’s anticancer activity across multiple cancer cell lines, coupled with mechanistic studies of cell cycle effects and cell death pathways. By revealing the distinctive chemical profile and selective anticancer properties of this honeybee-derived fungal strain, we seek to illuminate the therapeutic potential hidden within specialized ecological niches and advance our understanding of fungal natural products as promising sources of novel anticancer agents.

## Materials and methods

During our investigative screening process aimed at isolating fungi with anticancer properties from healthy bees, we successfully obtained a fungal isolate that demonstrated promising anticancer activity in preliminary tests.

### Characterization of the fungal isolate

Systematic sampling of honeybee specimens was conducted between October and December 2021 across ten apiaries in three Egyptian governorates: five in Dakahleya, one in Ismaelia, and four in Menoufia. This sampling strategy was designed to capture a representative diversity of bee populations. Healthy adult worker bees were collected from each apiary and transported to the laboratory in sterile containers for immediate processing. Surface sterilization of bee specimens was performed following the protocols of^24,25^ to eliminate external contaminants. The sterilization procedure involved sequential immersion in 2% sodium hypochlorite for 3 min, 70% ethanol for 60 s, 5% sodium chloride solution for 60 s, and a final rinse with sterile distilled water. After surface sterilization, three bees were aseptically crushed on the surface of each sterile agar plate to release internal microorganisms. For fungal isolation, Potato Dextrose Agar (PDA) and Chloramphenicol Rose Bengal Agar (both from Merck, Germany) were used. Hyphal tips from emerging colonies were picked with a sterile needle and transferred to fresh PDA plates for further purification.

The fungal isolate was morphologically and genetically characterized following standardized methods by^26^. Cultivation occurred on various media including Potato Dextrose Agar (PDA) and Czapek yeast autolysate (CYA) agar at 25 °C for 7 days, after which colony characteristics (color, reverse pigmentation, texture, appearance) were documented. Creatine sucrose agar (CREA) cultivation was employed to assess acid/base production. Microscopic examination was conducted on 7-day PDA cultures to observe the different structures as sporangiophores, sporangia, and sporangiospores. Genetic identification utilized the Gene Jet genomic DNA purification Kit for DNA extraction. Internal transcribed spacer (ITS) rDNA sequences were PCR-amplified using specific forward (5’-GACTCCTTGGTCCGTGTT-3’) and reverse (5’-TGAAATTGTTGAAAGGGAA-3’) primers via BIO-RAD T100 thermal cycler. The PCR product underwent purification with Gene JET PCR Purification Kit before sequencing with ABI Prism 3730XL DNA analyzer. The resulting nucleotide sequence received a GenBank accession number and was compared against published ITS rDNA sequences using BLAST to generate phylogenetic data. For phylogenetic analysis, the ITS rDNA sequence of our isolate was compared with reference sequences retrieved from the NCBI database using BLAST. The phylogenetic tree was constructed using the neighbor-joining method with the NCBI online tool. The reference strain used for comparison was *Aspergillus niger* [GenBank accession number: NR_111348].

### Fermentation and separation of the fungal crude extract

The process was initiated by the preparation of an inoculum, which is accomplished by cultivating the fungal isolate on potato dextrose agar medium (PDA) (Merck, Germany) at 28 °C for 7 days. Following that, a sterile cork borer was employed to cut a disc of 6 mm in diameter of PDA medium containing the fungal mycelium. This inoculum was subsequently transferred to fermentation 500 flasks containing 100 ml of potato dextrose broth medium (pH 5.6 ± 0.2). These flasks were incubated at 28 °C for 10 days under static conditions. Upon completion of the incubation time, the biomass was separated from the liquid medium via filtration which followed by centrifugation at 5000 rpm to eliminate the planktonic cells and obtain cell free filtrate (CFF). The CFF underwent a careful twice extraction process using 100% ethyl acetate (EtOAc) in a 1:1 ratio, agitated on a vortex shaker for 10 min, and allowed to settle for 5 min until distinct layers formed (organic layer and aqueous layer). The organic layer was separated using a separating funnel and concentrated under reduced pressure (40–45 °C) in a rotary evaporator (Heidolph VV2001, Germany) to yield a dark reddish-brown liquid. The crude extract was stored at -20 °C for subsequent analyses^27,28^.

### Characterization of the fungal crude extract

The crude extract was characterized via Gas chromatography-mass spectroscopy (GC–MS) and ultra-performance liquid chromatography (UPLC-MS/MS) analysis. The preparation of each sample was carried out by refluxing with 100 ml of absolute methanol and 5 ml sulphuric acid for 2 h, extracting with ether and drying the ethereal layer over anhydrous sodium sulfate followed by evaporation of ether to give residue, kept for GC-MS analysis^10^.

### GC-MS analysis

GC-MS analysis was performed using a Thermo Scientific Trace GC-ISQ mass spectrometer equipped with an A3000 auto sampler and a TG-5MS capillary column (30 m × 0.25 mm i.d. × 0.25 μm film thickness). The temperature was scheduled in a gradient mode (10 °C/minute) between 50 and 280 °C. Source temperature: 200 °C; interface temperature: 220 °C; injector temperature: 220 °C. Mass spectrometer adjusted in EI mode at 70 eV. One µL of diluted material was injected in splitless mode with a 50–600 amu mass scan. Helium was employed as the carrier gas (1 mL/min). A combination of fatty acid methyl esters (C5-C20) was immediately fed into the GC injector under the aforesaid temperature program to compute the retention index of each molecule for the identification of the components based on their retention indices. A comparison of the component’s mass spectral fragmentation patterns and base peak with those found in the literature or in the mass spectral databases NIST and ChemStation data system further corroborated the identity of the components^29^.

### UPLC-MS/MS analysis

Component identification was performed using a Waters UPLC XEVO TQD triple quadrupole mass spectrometer (Waters Corporation, Milford, MA 01757, USA). The chromatographic system consisted of a Waters Acquity QSM pump, an LC-2040 autosampler, a degasser, and a Waters Acquity CM detector. Chromatographic separation was achieved using a Waters Acquity UPLC BEH C18 column (50 mm × 2.1 mm i.d. × 1.7 μm particle size). The column was operated at a flow rate of 0.2 mL/min, and the system was thermostated at 30 °C to maintain optimal separation conditions. Mobile phase was composed of two phases. Phase A was composed of water + 0.1% formic acid, while phase B consisted of acetonitrile + 0.1% formic acid. Elution was a gradient one and its program was as following: 0.0–2.0 min, 10% B; 2.0–5.0 min, 30% B; 5.0–15.0 min, 70% B; 22.0 min, 90% B; 22.0–25.0 min, 90% B; 26.0 min, 100% B; 26.0–29.0 min, 100% B; 30.0 min, 10% B; followed by 4 min to re-equilibrate the column. The analysis was carried out using a triple quadrupole (TQD) mass spectrometer coupled to electrospray ionization (ESI) source. ESI in negative mode was operated at the following conditions: the capillary and cone voltages were set at 3 kV and 30 V, respectively. The ion source temperature was set at 150 °C and the pressure of the nitrogen gas (nebulizer) was adjusted at 35 psi. The temperatures of drying and sheath gas (N2) were 440 and 350 °C, respectively. The flow rates of the drying and sheath gas were adjusted at 900 and 50 L/h, respectively. Data were processed and analyzed using MZmine for peaks detection and alignment. Identified peaks were then matched against MassBank and mzCloud spectral databases to support compound identification. These tools facilitated the annotation of several metabolites detected in the sample^30^.

### In vitro cytotoxicity assessment of fungal extract on cancer and normal cell lines

All cell lines used in this study—including Caco-2, MCF-7, HepG-2, A549 (cancer cell lines), and WI-38, HFB-4 (normal cell lines)—were obtained from the cell culture bank at the Holding Company for Biological Products and Vaccines (VACSERA), Agouza, Egypt. Cells were cultured in Dulbecco’s Modified Eagle Medium (DMEM; Gibco) supplemented with 10% fetal bovine serum (FBS; Gibco), 1% penicillin-streptomycin, and maintained at 37 °C in a humidified atmosphere with 5% CO₂. Cells were subcultured every 2–3 days using 0.25% trypsin-EDTA when they reached approximately 80% confluence. All experiments were performed on cells in the logarithmic growth phase. The cytotoxic effects of fungal extract were evaluated in vitro against four cancer cell lines (Caco-2, MCF-7, HepG-2, and A549) and two normal cell lines (WI-38 and HFB-4) according to the methodology described by^31^.

To assess morphological changes, cells were seeded in 12-well plates at a density of 5 × 10⁵ cells/well in DMEM supplemented with 10% fetal bovine serum. The fungal extract was first dissolved in DMSO to prepare a stock solution, and working concentrations were obtained by serial dilution in culture medium. The final DMSO concentration in all wells, including controls, did not exceed 0.1% (v/v). The experiment was conducted in triplicate, with cell viability and proliferative potential determined using the MTT assay, which measures cellular metabolic activity. For the MTT assay, cancer cell lines were treated with the extract at concentrations of 0, 1.95, 3.9, 7.81, 15.62, 31.25, 125, 500, and 1000 µg/ml, while normal cell lines were exposed to 31.25, 125, 500, 1000, and 2000 µg/ml. After 24 h of incubation, morphological alterations were observed using phase-contrast microscopy (Olympus, Germany) and compared to untreated control cells. Doxorubicin, a chemotherapeutic agent, was used as a positive control at the same concentrations as the fungal extract.

### Live/dead cell viability visualization using confocal microscopy

Cells were cultured in a controlled environment at 37 °C, with 95% humidity and 5% CO2, using DMEM medium supplemented with growth factors and 10% fetal bovine serum (FBS). To ensure optimal cell density for imaging, cells were seeded onto glass-bottom dishes at a density of 1 × 10^5^ cells/mL. All cells were treated with IC50 value of the fungal extract. After a 24-hour incubation period, the treated and untreated (control) cells were subjected to live/dead staining dyes. For the live/dead assay, cells were incubated with Acridine Orange (AO) and Propidium Iodide (PI) for 15 min. AO stains both live and dead cells, while PI only enters cells with compromised membranes, thus staining dead cells. Following staining, cells were washed three times with phosphate-buffered saline (PBS) to remove excess dyes and minimize background fluorescence^32^.

### Cell cycle analysis

Cell cycle progression analysis following treatment with fungal extracts was performed using flow cytometry with propidium iodide (PI) staining. This technique quantifies cellular DNA content through PI’s stoichiometric intercalation with DNA, enabling cell cycle phase distribution determination. Cells were treated with the IC_50_ value of the fungal extract as determined by the MTT assay for each respective cell line multiple cell lines (MCF7, HepG2, Caco2, and A549) were examined to elucidate the extract effects according to the procedure described by^33^. with minor modification. Adherent cells were harvested by aspirating culture medium, washing with PBS, trypsinizing (37 °C, 2–5 min), and neutralizing with serum-containing medium. Suspension cells were collected directly by centrifugation. Both cell types were pelleted (300–400 × g, for 5 min), resuspended in PBS or flow cytometry buffer, and filtered through a 40 μm strainer to eliminate aggregates. Cells were then washed in PBS, pelleted (500 × g for 5 min), and fixed by resuspending in 400 µL ice-cold PBS followed by slow addition of 800 µL ice-cold absolute ethanol. Fixed samples were stored at + 4 °C for a minimum of two hours (stable for up to four weeks). Prior to analysis, fixed cells were equilibrated to room temperature, gently resuspended, pelleted (500 × g, 5 min), and washed once with PBS. The pellet was resuspended in 200 µL PI + RNase Staining Solution and incubated at 37 °C for 20–30 min in darkness. After incubation, samples were placed on ice, gently resuspended, and analyzed by flow cytometry. Forward versus side scatter gating excluded debris and aggregates while PI fluorescence was collected in the FL2 channel using 488 nm excitation. Data analysis involved establishing appropriate gates to exclude debris and delineating DNA content regions (< 2 N, 2 N, 2–4 N, 4 N, >4 N) on histogram plots.

### Apoptosis analysis

Apoptosis was assessed using the Annexin V-FITC Apoptosis Detection Kit (BioVision), which detects phosphatidylserine translocation from the inner to outer cell membrane during early apoptosis. The protocol was applied to various cell lines treated with IC_50_ value of the fungal crude extracts in addition to the nontreated cells (control) following the method of^19^. Cells (1–5 × 10^5) were collected by centrifugation (300–400 × g, 5 min) following treatment. The pellet was resuspended in 500 µL of 1X Binding Buffer before adding 5 µL Annexin V-FITC and optionally 5 µL propidium iodide (50 µg/mL). This mixture was incubated at room temperature for 5 min in darkness. For adherent cells, gentle trypsinization and washing with serum-containing media preceded the staining procedure. Flow cytometric analysis was performed using 488 nm excitation with emission detection at 530 nm for Annexin V-FITC (FL1) and PI detection via the phycoerythrin emission channel (FL2). For microscopic visualization, stained cell suspensions were mounted on glass slides with coverslips, or cells grown directly on coverslips were inverted onto slides after staining. Optional fixation in 2% formaldehyde could be performed after Annexin V-FITC binding to prevent nonspecific binding. Under fluorescence microscopy with dual FITC/rhodamine filters, apoptotic cells displayed green membrane staining, while necrotic cells exhibited red nuclear staining with green membrane fluorescence.

### Statistical analysis

All experiments were performed in triplicate and results expressed as mean ± standard error. Statistical analyses were conducted using appropriate software packages with significance set at *p* < 0.05. Cell Viability and Cytotoxicity Assays: IC50 values were calculated using non-linear regression analysis with four-parameter logistic curve fitting. Selectivity indices (SI) were determined as the ratio of IC50 values between normal cells (Wi-38, HFB-4) and cancer cell lines. One-way ANOVA followed by Tukey’s post-hoc test was employed to compare IC50 values across different cell lines and fungal extracts. Flow Cytometric Apoptosis Analysis: Annexin V-FITC/PI staining data were analyzed using flow cytometry software with quadrant gating to distinguish viable, early apoptotic, late apoptotic, and necrotic cell populations. Fold-change calculations were performed by dividing treated values by corresponding control values.

## Results and discussion

### Identification and characterization of the fungal isolate (AW17)

The fungal isolate AW17 was subjected to morphological and molecular characterization to confirm its taxonomic identity. Morphological examination was conducted across multiple growth media, revealed distinctive growth patterns and microscopic features characteristic of the *Aspergillus* genus. On Potato Dextrose Agar (PDA), colonies initially appeared white but progressively developed into black mature colonies with a cottony texture, reflected the developmental stages of conidiogenesis. The reverse side displayed a pale-yellow coloration, with occasional radial fissures developing in the agar due to growth patterns. Growth characteristics varied significantly across different media compositions. On Czapek Yeast autolysate Agar (CYA), the isolate produced olive-colored colonies with white to cream reversed coloration and demonstrated robust growth, reached 7.5 cm in diameter within a five-day incubation period at 28 °C. In contrast, colonies on PDA exhibited a more compact growth pattern characterized by a dusty brownish-black to black appearance. The isolate’s growth on Creatine sucrose agar (CREA) resulted in significant yellow color conversion, indicated active acid production associated with creatine hydrolysis a biochemical feature consistent with *Aspergillus niger* strains.

Microscopic examination revealed conidiophores measuring 6.0–12.0 μm in diameter with lengths extending up to 1.1 mm, terminated in globose radiate vesicles ranged from 27.0 to 45.0 μm in diameter. These vesicles supported biseriate conidiogenous cells, with metulae and phialides covering most of the vesicle surface area. The phialides produced spherical, dull brown conidia measuring 3.0–5.0 μm in diameter, forming radiate conidial heads morphological features that align with the established characteristics of *Aspergillus niger*.

**Fig. 1:**
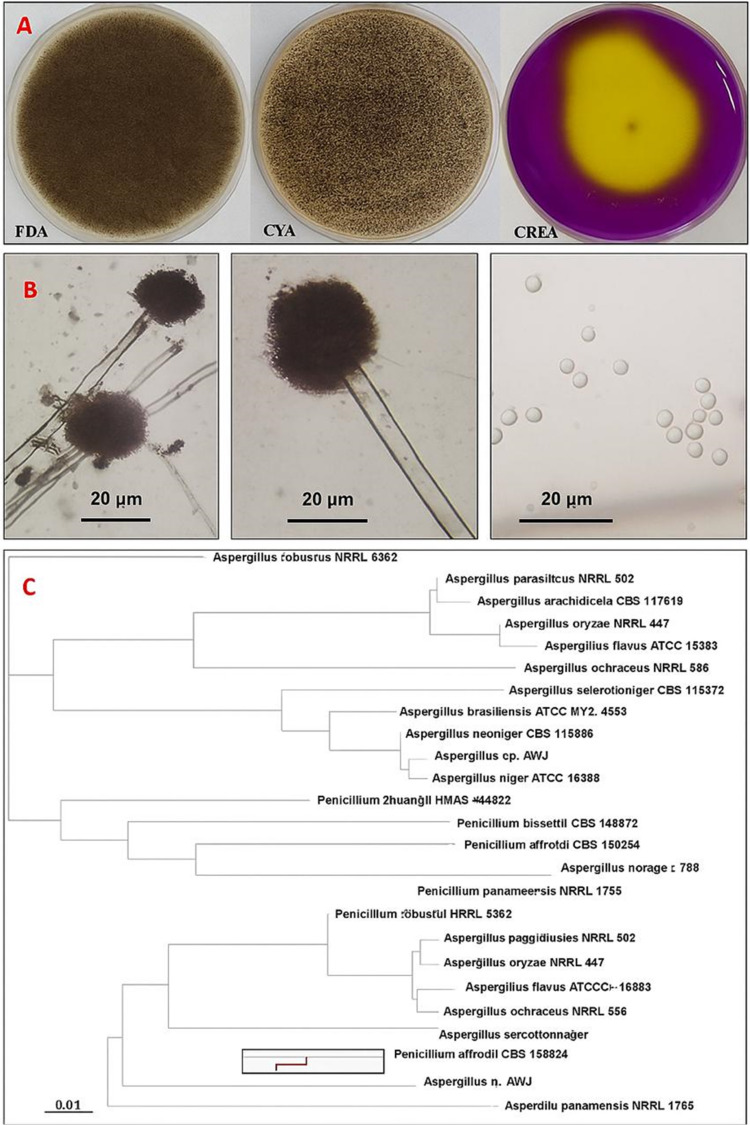
Morphological and molecular characterization of *Aspergillus niger* strain AW17 isolated from honeybees. (**A**) Macroscopic morphology of *A. niger* AW17 on different media. (**B**) Microscopic features of *A. niger* AW17 showing characteristic conidiophores with globose vesicles supporting biseriate conidiogenous cells (left and middle panels), and individual spherical conidia (right panel). (**C**) Phylogenetic tree based on ITS sequence analysis.

Molecular characterization of the *Aspergillus* AW17 isolate was performed using the Internal Transcribed Spacer (ITS) region. The obtained ITS sequence (377 bp) was subjected to multiple sequence alignment with reference sequences of *Aspergillus* species retrieved from the GenBank database. BLAST analysis revealed high sequence homology with *Aspergillus niger* strains, showing more than 99% sequence identity with reference sequences from the GenBank database. As shown in the phylogenetic tree (Fig. 1), the *A. niger* (AW17) isolate forms a distinct cluster with verified *A. niger* strains, demonstrating significant evolutionary distance from other *Aspergillus* species including *A. brasiliensis* (ATCC MYA-4553), *A. neoniger* (NRRL 502CS 115656), and other *Aspergillus* species included in the analysis. This molecular evidence, based on the highly discriminative ITS region, provides robust confirmation of the taxonomic identity of the isolate as *Aspergillus niger*, supporting the morphological characterization and establishing its phylogenetic position within the genus *Aspergillus*. The sequence of this fungal isolate was deposited in GeneBank under the name *Aspergillus niger* strain Aw17 with accession number PP907816.

Fungi represented important components of the honeybee microecosystem. It played vital ecological roles within hives, working variously as commensals, symbionts, opportunistic pathogens, and environmental constituents. The isolation of *A. niger* from honeybees aligns with previous studies recording the presence of diverse fungal taxa including *Aspergillus*,* Penicillium*,* Cladosporium*, and *Mucorales* in bee colonies. These fungi may entered the hive environment through multiple routes, including pollen collection, nectar foraging, or environmental exposure, subsequently establishing themselves within the hive matrix. The ecological role of *Aspergillus* species in honeybee colonies remained incompletely understood, potentially ranging from neutral environmental presence to more complex interactions with bee health and hive ecology^34^. The morphological features of strain AW17 match classic *A. niger* characteristics. The transition from white initial growth to black mature colonies on PDA, the olive-colored growth on CYA, and the notable acid production on CREA medium (visible as yellow color change) are all typical traits of *A. niger*. These features help distinguish it from closely related *Aspergillus* species. Microscopic examination revealed defining structures characteristic of *A. niger*, including the distinctive biseriate arrangement of conidiogenous cells on globose vesicles and the spherical brown conidia forming radiate heads. These microscopic details align perfectly with standard descriptions of *A. niger* in taxonomic literature. Molecular analysis using ITS sequencing provided definitive confirmation, showing over 99% sequence homology with reference *A. niger* strains. The phylogenetic analysis placed strain AW17 in a distinct cluster with verified *A. niger* strains, clearly separated from other *Aspergillus* species. This agreement between morphological, cultural, and genetic data provides strong evidence for accurate identification^35^. The isolation and characterization of *A. niger* strain AW17 from honeybees contributed to our understanding of the fungal component of the bee microbiome. *A. niger* is known for producing various enzymes and compounds that helped it utilize different food sources, which may explain its ability to survive in the hive environment^36,37^.

### Fermentation and characterization of *Aspergillus niger* crude extract

After the extraction process, the fermentation batch of *Aspergillus niger* was gave a yield of 1.3 g/l of crude extract. This crude extract was characterized as follows:

### GC/MS analysis

**Fig. 2:**
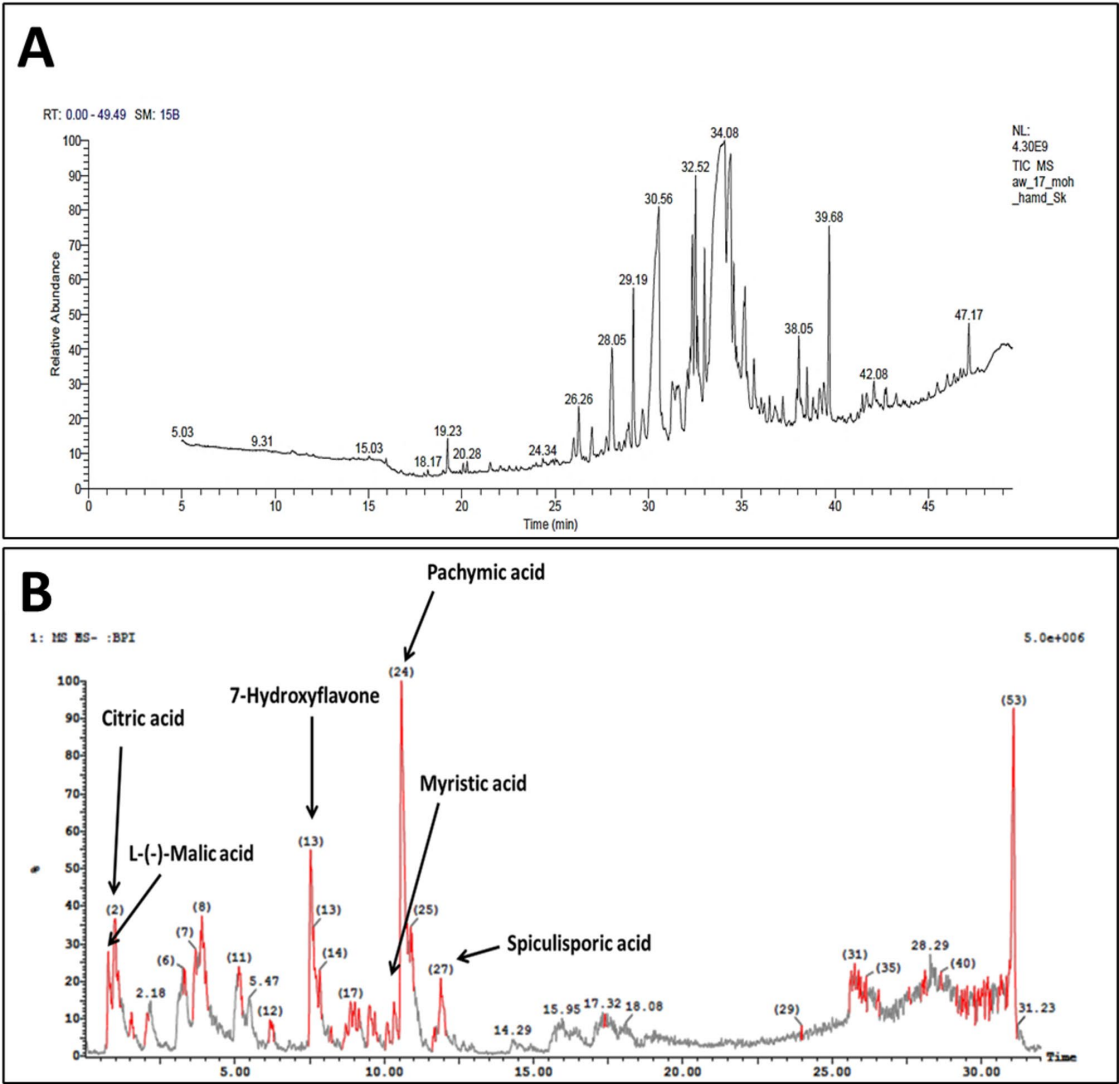
Characterization of *Aspergillus niger* extract; (**A**) Gas chromatogram, (**B**) UPLC-MS/MS chromatogram.

*Aspergillus niger* extract was subjected to GC-MS analysis, which yielded a complex combination of bioactive chemicals (Table S1). These compounds were further identified and separated on the basis of their mass spectra, retention times (Rt), and retention index (RI). A chromatogram, which was a graphical depiction of the GC-MS data, showed many peaks, each of which represented a different component in the sample. The time it took for each component to pass through the GC column and get at the detector is represented by the retention time, which is expressed in minutes. *Aspergillus niger* gas chromatography mass spectrometry identified sixty substances. Among the chemicals found, fatty acids and their esters make up a significant portion. The main constituents of fatty acids are hexadecanoic acid, oleic acid, and octadecanoic acid (stearic acid); the main constituents of fatty acid esters are methyl hexadecanoate, methyl oleate, and methyl linoleate (Table 1; Fig. 2A). After octadecanoic acid and hexadecanoic acid, the peak area, which showed the relative concentration of each constituent in the sample, showed that oleic acid is the most prevalent compound in the extract.

**Table 1:**
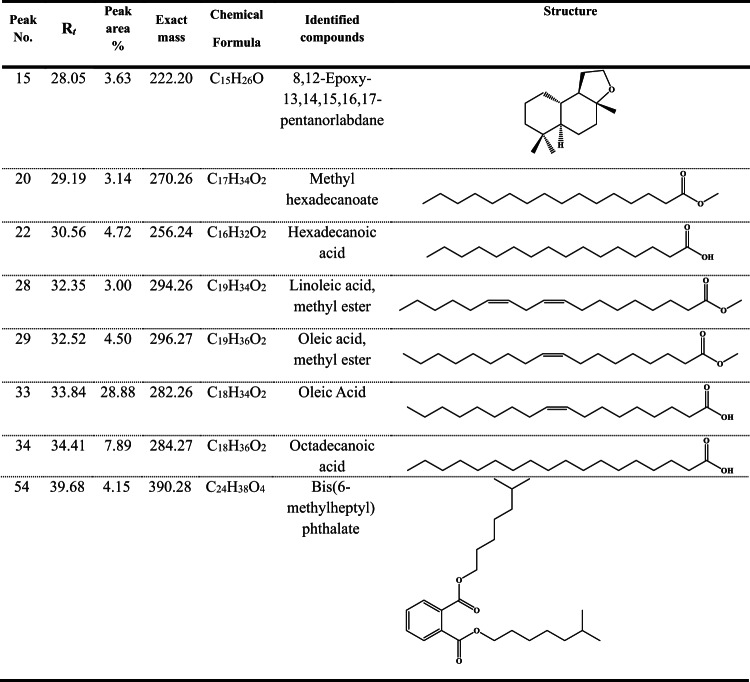
Major components of Aspergillus Niger as revealed by GC/MS analysis.

### UPLC-MS/MS

The ultra-performance liquid chromatography-tandem mass spectrometry (UPLC-MS/MS) analysis of the *Aspergillus niger* extract provided unequivocal evidence for the presence of fatty acids, with the identification of myristic acid serving as a paradigmatic example. Furthermore, this analytical approach also revealed the existence of six major compounds with varying retention times and molecular masses. At early retention times, two organic acids were detected: L-(-)-malic acid (Rt = 0.75 min) and citric acid (Rt = 0.98 min). At intermediate retention time, 7-hydroxyflavone was identified (Rt = 7.53 min), followed by myristic acid (Rt = 9.50 min). Two additional compounds were detected at later retention times: pachymic acid (Rt = 10.57 min) and spiculisporic acid (Rt = 11.89 min) as depicted in Table 2; Fig. 2B. These compounds represented diverse chemical classes including organic acids, flavonoids, fatty acids, and terpenoids present in the fungal extract.

**Table 2:**
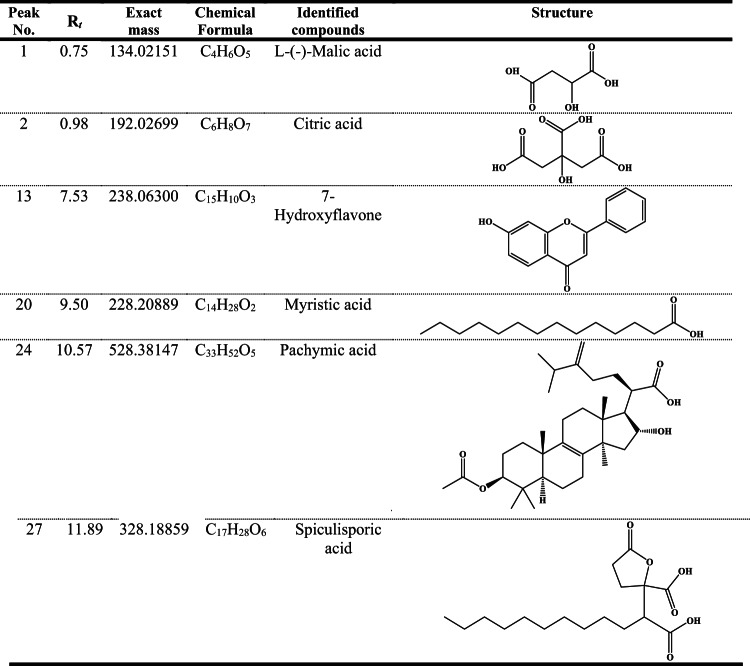
Major components of *Aspergillus Niger* as revealed by UPLC-MS/MS analysis.

The chemical profiling of *Aspergillus niger* crude extract through complementary analytical techniques (GC-MS and UPLC-MS/MS) revealed a complex biochemical composition dominated by several classes of bioactive compounds with potential therapeutic applications. The GC-MS analysis identified a predominant fatty acid signature, with oleic acid (28.88%) emerging as the most abundant constituent, followed by octadecanoic acid (7.89%) and hexadecanoic acid (4.72%). This fatty acid triad represented a characteristic profile frequently observed in *Aspergillus niger* extracts. The high proportion of oleic acid (C18:1) is particularly remarkable as it suggested significant metabolic investment in unsaturated fatty acid biosynthesis, potentially conferring membrane fluidity advantages to the organism^11^. The presence of these fatty acids aligned with their documented biological activities, including antimicrobial, antioxidant, and anticancer properties^38,39^. Particularly, the high oleic acid content (28.88%) exceeds typical concentrations reported in most *A. niger* strains, which generally range from 10 to 20%^40^. This elevated concentration might have reflected specific adaptations to the honeybee environment, possibly relating to nutritional availability or stress response mechanisms within the hive microenvironment. The detection of methyl esters (methyl hexadecanoate, methyl oleate, and methyl linoleate) at notable concentrations (3.14%, 4.5%, and 3.0%, respectively) might have indicted active fatty acid modification pathways. Regardless, these compounds demonstrate independent bioactivities as reported by^41^, potentially contributing to the overall biological activity of the extract. The predominance of fatty acids and their esters aligns with previous study on *A. niger* metabolites^42^ confirming established metabolic patterns across different strains. The identification of 8, 12-epoxy-13, 14, 15, 16, 17-pentanorlabdane (3.63%) represents one of terpenoid derivatives which known to have anti-breast cancer activity^43^. This labdane-type diterpenoid structure might have indicted the activation of the mevalonate pathway in this *A. niger* strain. This particular labdane diterpenoid has been rarely reported in *A. niger*, signifying possible strain-specific metabolic pathways or environmental adaptation mechanisms unique to this honeybee-associated isolate. The detection of bis(6-methylheptyl) phthalate (4.15%) raises considerations about its origin, as phthalates can be natural metabolites but are also common laboratory contaminants^44^.

The UPLC-MS/MS analysis further confirmed terpenoid biosynthesis through the detection of pachymic acid (Exact mass = 528.38147 Da), a lanostane-type triterpenoid with known bioactivity. This finding is particularly significant as it demonstrates *A. niger*’s capacity to produce complex secondary metabolites beyond primary metabolism. The detection of pachymic acid represents a significant deviation from typical *A. niger* metabolite profiles, as this compound has been primarily associated with medicinal mushrooms, particularly *Poria cocos*^45^. This unusual metabolic capability might have suggested unique biosynthetic pathways in this honeybee-derived strain, potentially acquired through horizontal gene transfer or developed through specific ecological adaptations. The UPLC-MS/MS analysis revealed significant organic acid production, notably citric acid (192.02699 Da) and malic acid (134.02151 Da). These organic acids represent primary metabolites from the tricarboxylic acid cycle and are consistent with the well-documented acidogenic nature of *A. niger*^46^. This acidogenic profile supports the observed yellow color conversion on CREA medium reported in the morphological characterization, providing corroborating evidence for the fungal identification and metabolic behavior. Citric acid had interesting biological action as a preservative due to its antibacterial characteristics. Moreover, it has intriguing anti-allergy, hyaluronic acid synthesis promotion, and melanin activity suppression properties^47^. Research on citric acid has shown that it can stop the development of cancer cells by causing apoptosis and reducing cell division^48^. Myristic acid detection complements the fatty acid profile established by GC-MS analysis, increasing significant fatty acid metabolism in this isolate. This compound might have contributed to membrane structure and may influence the extract’s cytotoxic effects through membrane disruption. Spiculisporic acid, a γ-butenolide compound produced previously by certain *Aspergillus* species and exhibited notable surfactant properties and antimicrobial activity^49^. Additionally, the molecular structure of spiculisporic acid contributes to its surface activity. Its amphiphilic nature allows it to interact with and disrupt cell membranes enhancing its biological efficacy. The presence of 7-hydroxyflavone is uncommon in fungi, however a recent study reported the microbial synthesis of 7-hydroxyflavone by *Amycolatopsis* sp. HSN-02, demonstrating its antimicrobial efficacy and potential biocontrol properties against plant pathogens^50^. This suggested that *Aspergillus niger* AW17 might have possessed the metabolic capability to synthesize or modify flavonoid compounds, which might have contributed to oxidative stress protection within the honeybee microenvironment. The divergences from typical *A. niger* metabolic profiles observed in this study might have reflected the unique ecological niche of this strain. Isolation from honeybees suggested environmental adaptations that could significantly influence secondary metabolite production. In summary, our study demonstrates that the honeybee-derived *Aspergillus niger* AW17 strain produces a unique array of bioactive compounds, including several that are rarely or not previously reported in traditional GC-MS and UPLC-MS/MS studies of *A. niger*. Notably, compounds such as pachymic acid, 8, 12-epoxy-13, 14, 15, 16, 17-pentanorlabdane, 7-hydroxyflavone, and spiculisporic acid distinguish the metabolic profile of this isolate from those of strains obtained from other environments. These findings underscore the novelty of our work and highlight the value of exploring specialized ecological niches such as the honeybee microbiome for the discovery of new fungal metabolites with potential therapeutic applications^51^.

### Cytotoxicity of the *Aspergillus niger* extract against cancer and normal cell lines

**Fig. 3:**
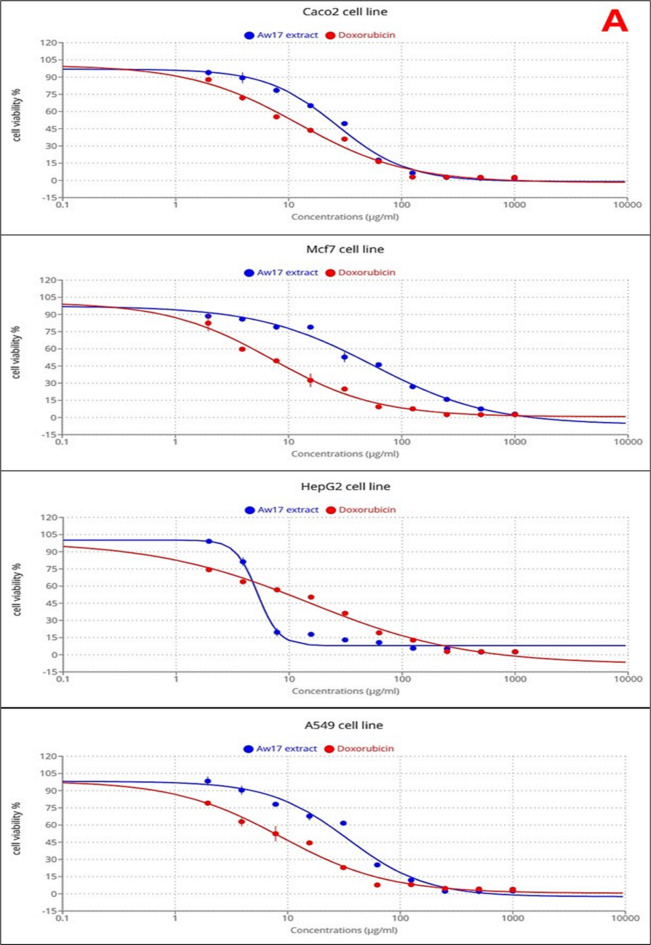

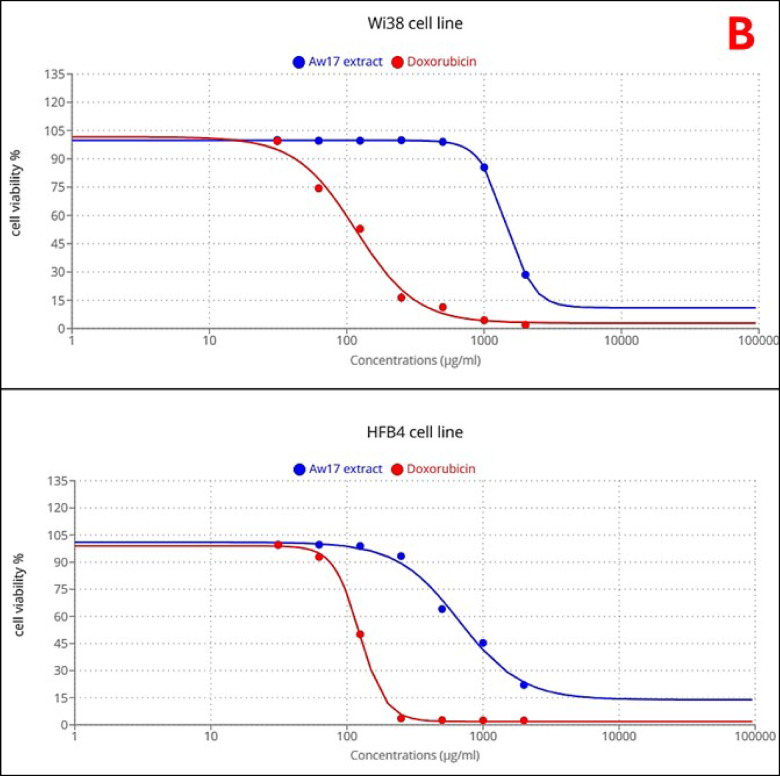
Cytotoxic and selective effects of honeybee-derived Aspergillus niger AW17 extract. (**A**) Dose-dependent inhibition of viability in human cancer cell lines (Caco-2, MCF-7, HepG-2, A549) compared to doxorubicin, as determined by MTT assay. (**B**) Relative resistance of normal cell lines to the extract at lower concentrations, demonstrating high selectivity for cancer cells.

The fungal extract demonstrated significant dose-dependent cytotoxicity across all tested cancer cell lines: Caco-2 (colorectal adenocarcinoma), MCF-7 (breast cancer), HepG-2 (liver cancer), and A549 (lung cancer), with notable variability in sensitivity profiles compared to the positive control doxorubicin (Fig. 3A). HepG-2 cells exhibited the highest susceptibility to the fungal extract, characterized by a quick decline in viability within the 3–10 µg/ml concentration range and an IC_50_ value of 5.22 µg/ml, demonstrating superior sensitivity compared to doxorubicin (IC_50_ = 15.13 µg/ml). Caco-2 cells demonstrated the second highest sensitivity to the fungal extract with an IC_50_ of 26.78 µg/ml, though doxorubicin exhibited superior cytotoxicity with an IC_50_ of 12.18 µg/ml, showing approximately 2-fold greater potency. A549 cells displayed moderate sensitivity to the fungal extract (IC_50_ = 34.18 µg/ml), while doxorubicin demonstrated markedly superior activity with an IC_50_ of 8.79 µg/ml, representing nearly 4-fold greater potency. MCF-7 cells exhibited the greatest resistance to the fungal extract among all cancer cell lines tested, requiring substantially higher concentrations to achieve cytotoxicity with an IC_50_ of 55.91 µg/ml. Doxorubicin showed dramatically superior activity against MCF-7 cells with an IC_50_ of 7.19 µg/ml, demonstrating approximately 8-fold greater potency than the fungal extract. In contrast to the cancer cell lines, both normal cell lines exhibited remarkable resistance to the fungal extract at lower concentrations, demonstrating excellent selectivity (Table 3; Fig. 3B). WI-38 cells maintained high viability until higher concentrations were reached, with an IC_50_ of 1454.7 µg/ml for the fungal extract, while doxorubicin showed significantly greater cytotoxicity against this normal cell line with an IC_50_ of 116.27 µg/ml. HFB-4 cells demonstrated similar resistance patterns with an IC_50_ of 668.3 µg/ml for the fungal extract and 123.9 µg/ml for doxorubicin Table 3. The selectivity analysis revealed a striking difference between the two treatments. The fungal extract demonstrated exceptional selectivity for cancer cells over normal cells with a mean Selectivity Index (SI) of 73.26 while doxorubicin showed a substantially lower mean SI of 12.04.

**Table 3:** IC_50_ values (µg/ml) of Aspergillus niger extract and doxorubicin against human cancer and normal cell lines.

	Agent cancer	Origin cell	IC_50_ (µg/ml) extract	IC_50_ (µg/ml) doxorubicin
Cancer cell line	HepG2	Liver cancer	5.22	15.13
	Caco-2	Colorectal cancer	26.78	12.18
	A549	Lung cancer	34.18	8.79
	McF7	Breast cancer	55.91	7.19
Normal cell line	Wi-38	Lung fibroblast	1454.7	116.27
	HFB-4	Skin fibroblast	668.3	123.9

**Fig. 4:**
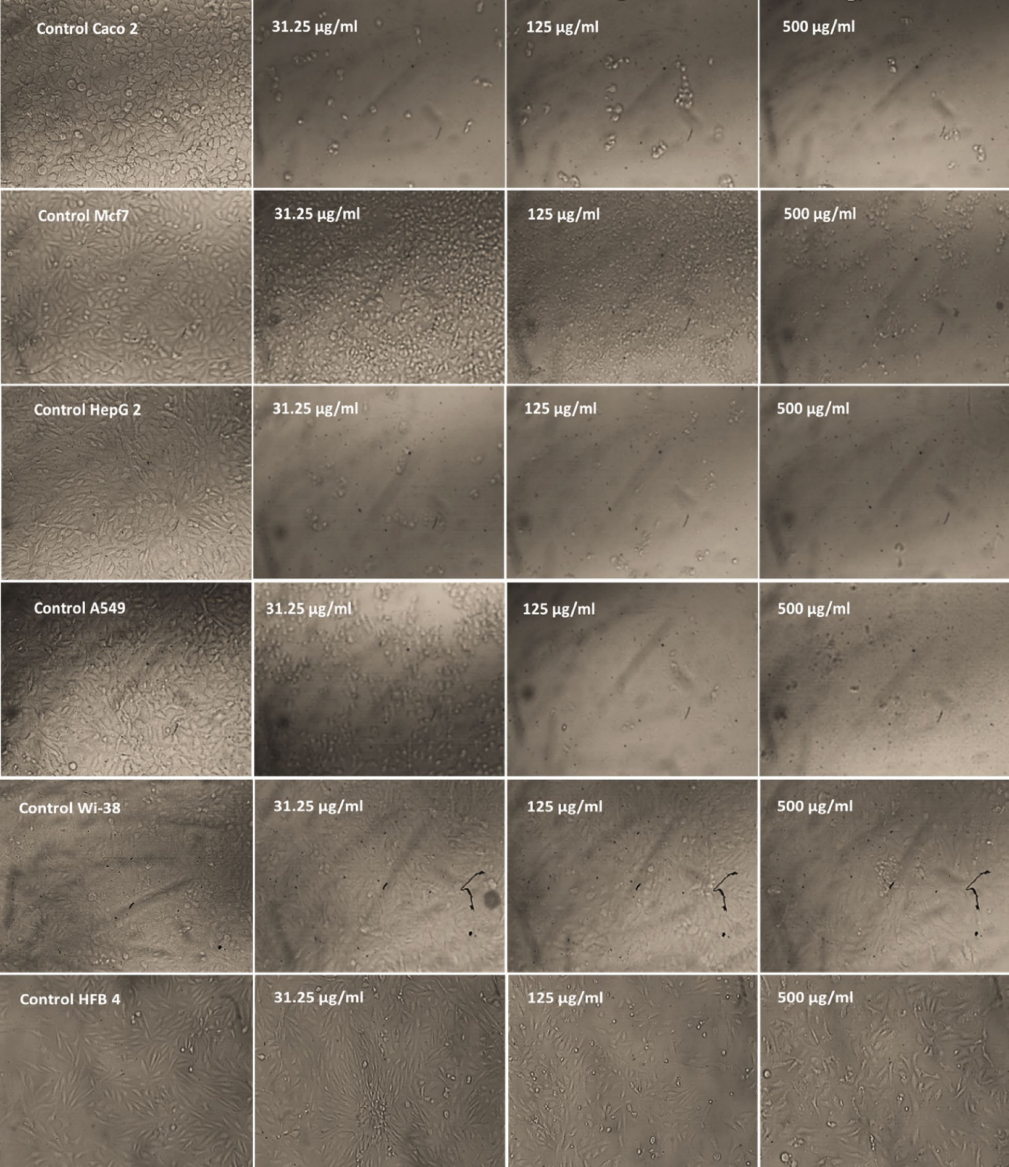
Morphological observations in cancer and normal cell lines under inverted microscopy.

Inverted microscopy revealed progressive concentration-dependent morphological alterations across all cancer cell lines (Fig. 4). Control cultures displayed characteristic morphology with appropriate confluence and intact cellular structures. At 31.25 µg/ml, Caco-2 and HepG-2 cells exhibited early signs of cytotoxicity, including partial detachment, cytoplasmic vacuolization, and reduced cellular density. A549 and MCF-7 cells maintained near-normal morphology at this concentration, consistent with their higher IC_50_ values. At 125 µg/ml, obvious effects were observed across all cancer cell lines, characterized by substantial reductions in cell density and extensive cellular detachment. The 500 µg/ml treatment induced near-complete destruction of the cancer cell monolayers, with remaining cells appearing rounded and detached and extensive cellular debris. On the contrary, microscopic observations of normal cell lines revealed minimal morphological changes even at higher extract concentrations (Fig. 4). At 31.25, 125, and 500 µg/ml, both WI-38 and HFB-4 cells maintained their characteristic fibroblastic morphology with elongated, spindle-shaped appearances and adherent properties. There was minimal evidence of cellular detachment, rounding, or membrane blebbing that would typically indicate cytotoxicity, even at the 500 µg/ml concentration.

The *Aspergillus niger* extract demonstrated significant anticancer activity with superior selectivity compared to doxorubicin. While doxorubicin showed higher potency against most cancer cell lines, the fungal extract exhibited a 6-fold higher selectivity index (SI = 73.26 vs. 12.04), indicating substantially lower toxicity to normal cells. The extract’s superior activity against HepG-2 cells and variable sensitivity across different cancer types may be suggest selective cytotoxic mechanisms rather than general cellular toxicity. The distinct sensitivity order (HepG-2 > Caco-2 > A549 > MCF-7) proposes cell-type specific mechanisms of action, likely related to variations in cellular targets or metabolic processing of bioactive compounds. The exceptional sensitivity of HepG-2 and colorectal adenocarcinoma cells (IC_50_ =5.22 ,and 26.78 µg/ml, respectively) compared to the relatively resistant breast cancer cells (IC_50_ =55.91 µg/ml ) may indicate potential specificity for gastrointestinal malignancies. This selective cytotoxicity can be directly correlated with the extract’s chemical composition. The synergistic effect of high concentration of oleic acid and unique combination of the other bioactive compounds (labdane diterpenoids (8,12-epoxy-13,14,15,16,17-pentanorlabdane) and pachymic acid) in this honeybee-derived strain may explain the extract’s efficacy against specific cancer types while sparing normal cells^52,53^. These findings align with previous studies demonstrating *A. niger* metabolites’ anticancer potential, though with notable differences. Sajer et al.^54^, reported similar selective toxicity of *A. niger* compounds against hepatoma cell line (HepG2), colorectal cancer cell lines (HCT8) and (HCT116), human drug-resistant metastatic breast cancer cell line (MDA-MB-231) and epithelial-like breast carcinoma cell lines (KAIMRC1) and triple-negative (KAIMRC2) though with higher IC_50_ values 91.06, 193.5, 176.8, 98.46, 615.4 and 324.9 µg/ml, respectively. Moreover, our findings are consistent with recent studies involving other *Aspergillus* species. For example, *Aspergillus stellatus* LM-03 demonstrated anticancer activity against MCF-7 breast cancer cells, with an IC_50_ value of 33.24 µg/ml^55^. Similarly, Elkhouly et al.^56^ documented the crude extract from *Aspergillus tubingensis* ASH4 exhibited potent antiproliferative activity against colon, liver, and breast cancer cell lines, with IC_50_ values of 9.18 µg/well for colon cancer, 19.37 µg/well for hepatocellular carcinoma, and 5.89 µg/well for breast cancer.

### Differential cytotoxicity of *Aspergillus niger* extract on cancer cell lines using confocal microscopy

**Fig. 5:**
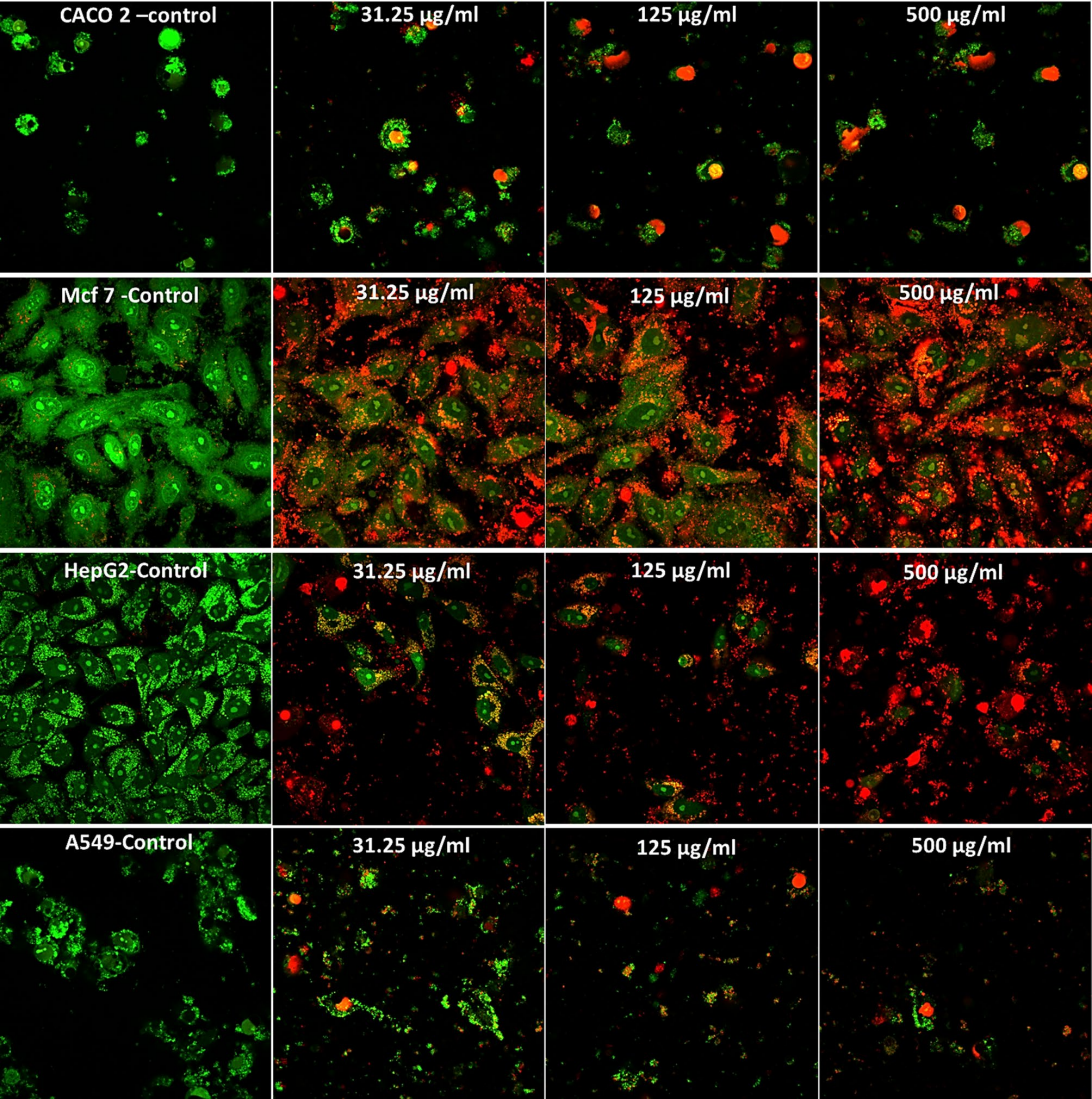
Dose-dependent cytotoxic effects of *Aspergillus niger* extract on CACO-2, MCF-7, HepG2, and A549 cancer cell lines. Confocal microscopy images of cells stained with acridine orange (green, viable cells) and propidium iodide (red, non-viable cells) after treatment with increasing concentrations (0, 31.25, 125, and 500 µg/ml) of *A. niger* extract for 24 h.

The confocal microscopy images stained with Acridine Orange and Propidium Iodide clearly demonstrated the differential effects of *Aspergillus niger* extract on four cancer cell lines at varying concentrations (Fig. 5). CACO-2 intestinal cancer cells exhibiedt extreme sensitivity to the fungal extract even at the lowest concentration (31.25 µg/ml), where a significant shift from green to red fluorescence is observed compared to the control, indicating substantial membrane damage. This effect intensified dramatically at higher concentrations (125 and 500 µg/ml), where predominant red fluorescence signifies widespread cell death and near-complete loss of membrane integrity across the cell population. HepG2 liver cancer cells also displayed high susceptibility to the extract, showing a progressive transition from primarily green fluorescence in the control to increasing red fluorescence as extract concentration rises. At 500 µg/ml, the vast majority of cells exhibited red fluorescence, demonstratied severe membrane compromise and extensive cell death, though slightly less pronounced than in CACO-2 cells. A549 lung cancer cells showed moderate sensitivity to the fungal extract, maintaining more viable (green) cells at lower concentrations compared to CACO-2 and HepG2. However, a clear dose-dependent increase in red fluorescence is still observed, particularly at 500 µg/ml, indicating that while more resistant than the aforementioned cell lines, these cells still undergo significant membrane damage at higher extract concentrations. MCF-7 breast cancer cells demonstrated the greatest resistance to the *Aspergillus niger* extract among all tested cell lines. Even at the highest concentration (500 µg/ml), these cells retained considerable green fluorescence, revealed that a substantial proportion of the cell population maintained membrane integrity despite exposure to high extract levels. Regarding concentration effects, all cell lines showed a positive correlation between extract concentration and cellular damage (increased red fluorescence), though with varying degrees of sensitivity. The 31.25 µg/ml concentration produced notable effects in CACO-2 cells, while achieving similar levels of damage in MCF-7 cells requires much higher concentrations. Based on these results, the cancer cell lines can be ranked according to their sensitivity to *Aspergillus niger* extract as follows: CACO-2 (most sensitive) > HepG2 > A549 > MCF-7 (most resistant).

The confocal microscopy analysis using Acridine Orange/Propidium Iodide dual staining provided compelling visual evidence of the differential cytotoxic effects of *Aspergillus niger* extract across the four cancer cell lines. These visual findings aligned precisely with the previously established cytotoxicity profile from viability assays. The confocal microscopy results not only confirm the sensitivity ranking (CACO-2 > HepG2 > A549 > MCF-7) established through viability testing but also provide insights into the mechanism of action. The shift from green to red fluorescence specifically indicated loss of membrane integrity as a primary cytotoxic effect, which might have explained the differential sensitivity observed. Cell-type specific responses to cytotoxic extracts likely arised from differences in membrane composition and repair mechanisms across cancer types. For example, drug-resistant non-small cell lung cancer cells showed faster membrane resealing due to annexin overexpression, increasing their resistance to membrane-targeting agents^57^. Similarly, colorectal cancer cell lines varied in sensitivity to cytotoxic compounds, linked to differences in membrane properties and repair capacity^58^. Alterations in membrane lipids, ion channels, and adhesion molecules affect drug uptake and resistance, while agents like phenothiazines disrupt membrane integrity, exploiting these susceptibilities^59^. These findings supported that membrane dynamics determined cancer cell susceptibility to cytotoxic extracts. This differential membrane permeabilization likely reflected fundamental differences in cellular biology between these cancer types. Gastrointestinal origin cells (CACO-2) typically expressed different membrane lipid compositions and transporter proteins compared to breast cancer cells (MCF-7), which might have accounted for their heightened susceptibility. Colorectal cancer cells often displayed altered membrane fluidity and reduced cholesterol content compared to other cancer types, potentially creating greater susceptibility to membrane-disrupting agents^60^. Additionally, digestive system cancers frequently exhibited reduced expression of multidrug resistance transporters that might otherwise expel cytotoxic compounds. This patternwas consistent with gradual accumulation of the bioactive compounds within cellular membranes until a critical threshold is reached, triggering catastrophic membrane failure. The presence of some cells maintained membrane integrity even at high concentrations, particularly in resistant lines like MCF-7, indicated potential heterogeneity within cancer cell populations, which may have significant implications for therapeutic strategies targeting these malignancies.

### Impact of *Aspergillus niger* extract on cell cycle distribution in cancer cell lines

**Fig. 6:**
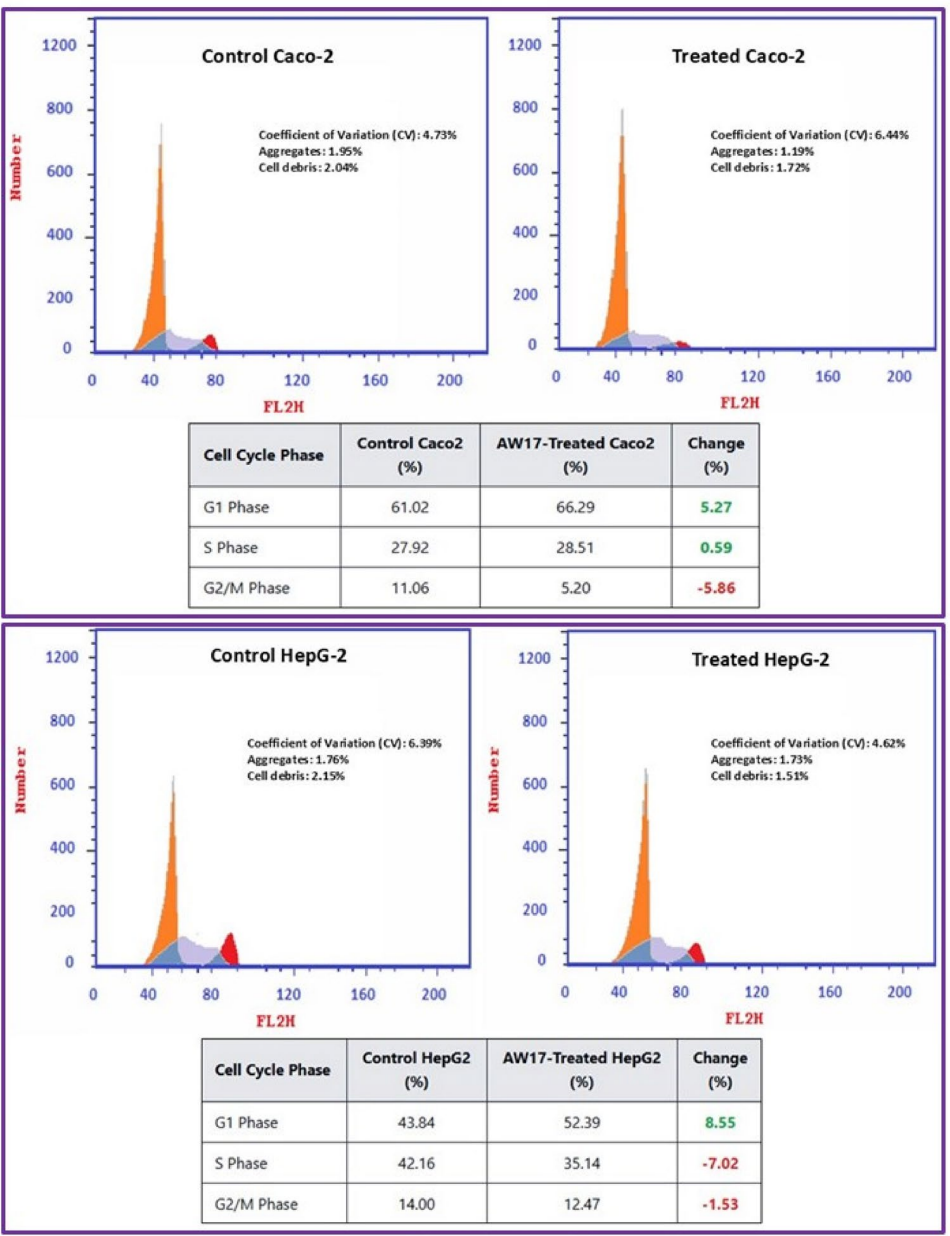

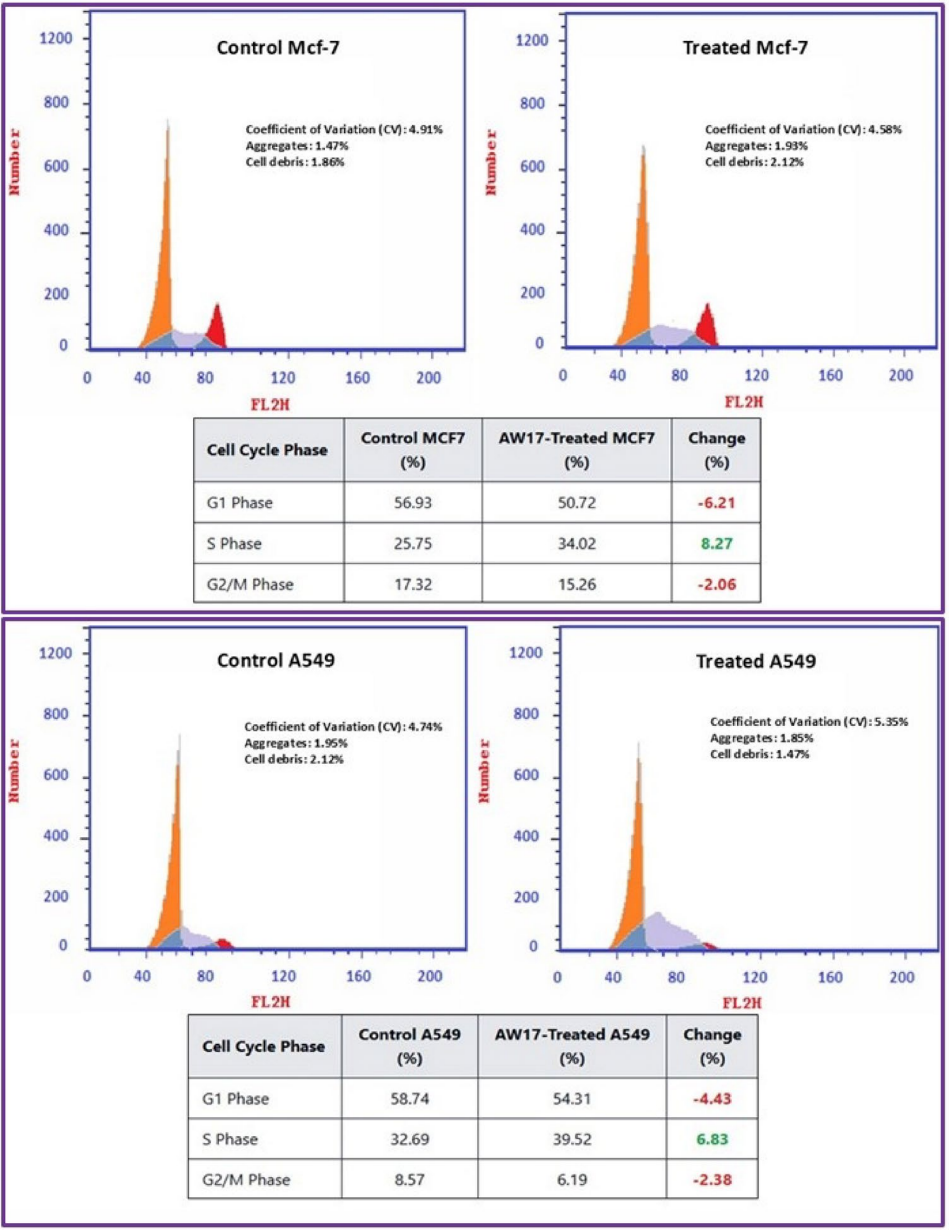
Effect of *Aspergillus niger* extract (AW17) on cell cycle distribution in four cancer cell lines. Flow cytometry histograms showing DNA content analysis of control and AW17-treated cancer cells with corresponding quantitative tables below each panel. Treatment induced differential cell cycle arrest patterns: G1 phase arrest in Caco-2 cells (61.02–66.29%, + 5.27%) and HepG2 cells (43.84–52.39%, + 8.55%), and S phase arrest in MCF-7 cells (25.75–34.02%, + 8.27%) and A549 cells (32.69–39.52%, + 6.83%). Green and red values in the tables indicate positive and negative changes, respectively.

The cell cycle analysis of four different cancer cell lines treated with *Aspergillus niger* extract (AW17) revealed distinct cell-type-specific responses with clear patterns of cell cycle arrest. Flow cytometry results demonstrated significant alterations in cell cycle phase distribution following AW17 treatment, providied insight into potential anticancer mechanisms (Fig. 6). In Caco-2 intestinal cancer cells, AW17 treatment induced a pronounced G1 phase arrest, evidenced by an increase in the G1 population from 61.02 to 66.29% (+ 5.27%). This G1 accumulation was accompanied by a marginal increase in S phase cells (27.92–28.51%) and a substantial reduction in G2/M phase cells (11.06–5.20%). The significant decrease in G2/M population (-5.86%) suggested that Caco-2 cells are primarily blocked at the G1/S checkpoint, preventing progression through the complete cell cycle. HepG2 liver cancer cells displayed a similar but more pronounced G1 phase arrest pattern. Treatment resulted in an 8.55% increase in G1 phase cells (43.84–52.39%), coupled with a substantial decrease in S phase population (42.16–35.14%, representing a -7.02% change). The G2/M phase showed a modest reduction from 14.00 to 12.47% (-1.53%). This profile indicated that AW17 effectively targets the G1/S checkpoint regulation in HepG2 cells, potentially interfering with key cell cycle regulatory proteins that control this transition point. In contrast, MCF-7 breast cancer cells exhibited a distinct S phase arrest pattern. AW17 treatment caused a significant reduction in G1 phase cells (56.93–50.72%, -6.21%) with a corresponding increase in S phase population (25.75–34.02%, + 8.27%). The G2/M fraction showed a slight decrease (17.32–15.26%, -2.06%). This pattern suggested that AW17 permits G1 to S progression but impedes DNA synthesis or completion of S phase, potentially through interference with DNA replication machinery or induction of replication stress. A549 lung cancer cells demonstrated a similar S phase arrest profile to MCF-7 cells. Treatment resulted in decreased G1 phase cells (58.74–54.31%, -4.43%) with a concurrent increase in S phase population (32.69–39.52%, + 6.83%) and reduction in G2/M phase cells (8.57–6.19%, -2.38%). This indicates that A549 cells respond to AW17 through mechanisms that permit G1 exit but hinder S phase completion.

### Flow cytometry analysis of apoptosis and necrosis in cancer cell lines treated with *Aspergillus niger* extract

**Fig. 7:**
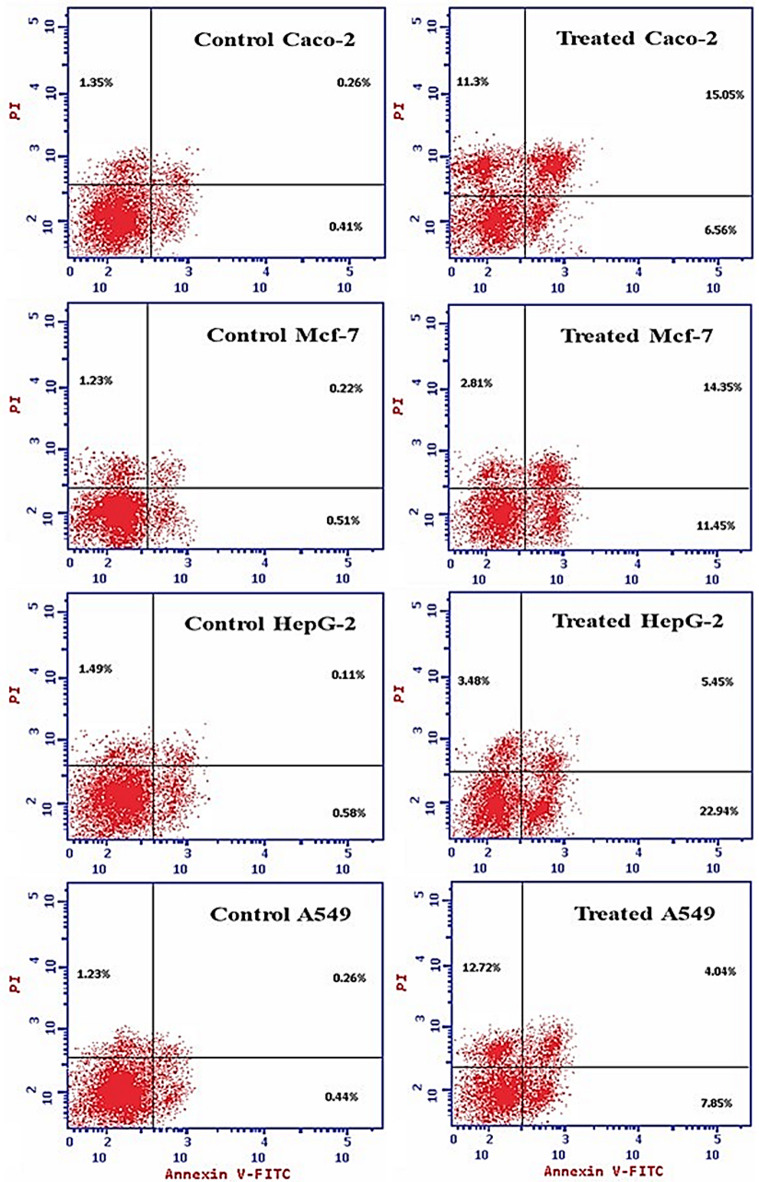
Flow cytometry analysis of apoptosis and necrosis in cancer cell lines following *Aspergillus niger* extract (AW17) treatment. Annexin V-FITC/PI dual staining showing the distribution of viable cells (lower left quadrant), early apoptotic cells (lower right quadrant), late apoptotic cells (upper right quadrant), and necrotic cells (upper left quadrant) in control and AW17-treated cancer cells. Treatment induced distinct cell death patterns across different cancer types: prominent early apoptosis and necrosis in Caco-2 cells (32.91% total cell death), predominant late apoptosis in HepG-2 cells (31.87%), balanced early and late apoptosis in MCF-7 cells (28.61%), and substantial necrosis in A549 cells (24.61%). Percentages in each quadrant represent the proportion of cells in the corresponding death state.

Flow cytometry analysis using Annexin V-FITC/PI dual staining revealed distinct cell death patterns across four cancer cell lines following *Aspergillus niger* extract treatment (Fig. 7). Control populations of all cell lines exhibited minimal baseline cell death (1.93–2.18%), confirmed appropriate experimental conditions for assessing treatment effects. Caco-2 intestinal cancer cells demonstrated the highest sensitivity to the fungal extract, with total cell death increasing from 2.02 to 32.91% post-treatment. The cell death profile in Caco-2 cells was characterized by prominent early apoptosis (15.05%) and substantial necrosis (11.3. HepG-2 liver cancer cells showed comparable general sensitivity with 31.87% total cell death, but displayed a distinctly different pattern dominated by late-stage apoptosis (22.94%). MCF-7 breast cancer cells exhibited moderate sensitivity with 28.61% total cell death following treatment. Their response pattern featured balanced distribution between early (14.35%) and late (11.45%) apoptotic stages with minimal necrosis (2.81%). A549 lung cancer cells showed the lowest general sensitivity (24.61% total death) with a unique death profile characterized by predominant necrosis (12.72%) with secondary contributions from apoptotic mechanisms.

The investigation of *Aspergillus niger* extract’s effects on cancer cells revealed a multifaceted cytotoxic mechanism involving both cell cycle disruption and programmed cell death induction. The observed pattern of differential sensitivity (Caco-2 > HepG2 > MCF-7 > A549) was consistently reflected across multiple analytical approaches, suggesting a clear mechanistic foundation underlying the extract’s anticancer activity. The cell cycle analysis revealed particularly intriguing cell-type specific responses, with Caco-2 and HepG2 cells exhibited G1 phase arrest, while MCF-7 and A549 cells demonstrated S phase accumulation. This dual pattern suggested the extract contains bioactive compounds capable of targeting different regulatory checkpoints depending on the specific molecular features of each cancer type. G1 arrest in gastrointestinal cancers involved cyclin-dependent kinase inhibition or suppression of G1/S transition proteins, while S phase arrest in the other cell lines likely reflected interference with DNA replication machinery^61–63^. These cell cycle perturbations appeared to be directly connected to the observed apoptotic and necrotic responses. The flow cytometry apoptosis data revealed that while general cell death percentages follow similar sensitivity rankings, the cell death mechanisms differ significantly. Caco-2 cells primarily undergo early apoptosis with substantial necrosis, while HepG2 cells predominantly displayed late apoptosis. This suggested the extract triggers different death pathways depended on where cells were arrested in their cycle. The G1-arrested cells (Caco-2 and HepG2) showed greater sensitivity to apoptosis induction, possibly reflected activation of intrinsic apoptotic pathways when cells fail to progress to DNA synthesis^64,65^. Intriguingly, the extract induced distinctly different death profiles in MCF-7 and A549 cells despite both showed S phase arrest. MCF-7 cells exhibited balanced early and late apoptosis with minimal necrosis, while A549 cells showed predominant necrosis. This divergence might have reflected differences in checkpoint response mechanisms, DNA damage repair capacity, or intrinsic apoptotic machinery between these cell types. Recent research supported this notion, showed that A549 lung cancer cells have impaired DNA repair and are more disposed to necrotic cell death under genotoxic stress compared to MCF-7 breast cancer cells, which favor apoptotic pathways^66,67^. These findings shared similarities with previous studies but offer unique insights. Jiang et al.^68^ reported G1 arrest in hepatocellular carcinoma treated with *Aspergillus*-derived compounds, consistent with our observations in HepG2 cells. Moon et al.^69^, reported that yeast extract was showed to induce G0/G1 phase arrest in colorectal cancer cells via activation of the p38-p53-p21 signaling cascade, leading to inhibition of proliferation and increased apoptosis. These results in accordance with our findings. However, Wu et al.^70^, found predominant G2/M arrest in colorectal cancer cells after treatment with taxol and nocodazole as anticancer agents, contrasting with the G1 arrest we observed in Caco-2 cells. Regarding cell death mechanisms, our findings align with^71^, who documented strain-dependent variability in apoptosis profiles across cancer types treated with fungal extracts. The distinct cell death profiles observed apoptosis in MCF-7 and HepG2, predominant necrosis in A549, and a mix in Caco-2 may be attributed to the interplay between the chemical composition of the AW17 extract and the intrinsic properties of each cancer cell line. Compounds such as oleic acid and pachymic acid have been shown to induce apoptosis via mitochondrial membrane depolarization, ROS generation, and activation of caspase cascades^11,72,73^. Labdane-type diterpenoids and other membrane-active metabolites can disrupt plasma membrane integrity, leading to necrosis, particularly in cells with defective apoptotic machinery^44,74,75^.

The induction of necrosis by the extract may offer therapeutic advantages, as necrotic cell death can enhance anti-tumor immune responses through the release of tumor antigens and DAMPs^76–78^. However, it is important to consider that excessive necrosis could also promote inflammation, which may be detrimental in certain tumor microenvironments. Thus, the ability of the AW17 extract to induce both apoptosis and necrosis may contribute to its broad-spectrum anticancer activity, but further mechanistic studies are warranted to optimize its therapeutic potential. Although our isolate is *Aspergillus niger*, a fungus typically considered terrestrial and not widely known for anticancer activity, we found that when it is isolated from honeybees as part of the bee microbiome, it shows unusual and promising anticancer effects. This opens a new door to isolating microbes from bees and exploring their biological activities. However, it is important to note that all our experiments were performed in vitro, using cancer cell lines under laboratory conditions. These results may not fully reflect what happens in the complex environment of a living organism, where many other factors can influence the outcome. Further studies, including animal experiments and investigations into the metabolic pathways involved, will be needed to confirm and better understand these findings.

## Conclusion and future prospects

This study highlights *Aspergillus niger* strain AW17, isolated from honeybees, as a promising source of selective anticancer agents. Its extract shows strong cytotoxicity against gastrointestinal cancers with minimal effects on normal cells, offering a broader therapeutic window than many known fungal extracts. The strain’s unique metabolite profile marked by high oleic acid and rare compounds like pachymic acid likely reflects its adaptation to the honeybee microenvironment. Mechanistically, the extract induces cancer-type specific responses: G1 arrest and apoptosis in gastrointestinal cancer cells, and S phase arrest with diverse death pathways in respiratory and mammary cancer cells. These effects suggest the involvement of multiple synergistic bioactive compounds targeting different cellular processes. The unique chemical profile and selective anticancer activity of the honeybee-derived *Aspergillus niger* strain AW17 reflect its ecological adaptation and reveal promising potential for targeted cancer therapies showing once again that nature, especially where diverse organisms interact, remains a powerful source of novel and effective therapeutic compounds.

Future work should command the isolation and characterization of active terpenoids and fatty acids to clarify their individual roles. In vivo testing is essential to validate therapeutic potential. The extract’s selective membrane-disrupting activity also warrants deeper investigation as a foundation for developing targeted, low-toxicity cancer therapies. Additionally, exploring the ecological role of these metabolites within the hive microbiome could reveal novel microbial interactions.

## Supplementary Information

Below is the link to the electronic supplementary material.

Supplementary Material 1

## Data Availability

“The datasets generated during and/or analysed during the current study are available in the GeneBank under the name *Aspergillus niger* strain Aw17 with accession number PP907816.”
