# Supplementary material for: Honeybee associated Aspergillus niger AW17 as a source of selective anticancer compounds with cytotoxicity evaluation in human cancer cell lines
Source: Sci Rep. 2025 Sep 12;15:32472. doi: 10.1038/s41598-025-18565-y (PMC12432222; doi:10.1038/s41598-025-18565-y)
Supplement: Supplementary file 1 — Supplementary Material 1 [file 41598_2025_18565_MOESM1_ESM.docx]

Table S1 Major components of Aspergillus niger as revealed by GC-MS analysis.

| **Peak No.** | **R*_t_*** | **R*_I_*** | **Peak area %** | **Identified compounds** |
| --- | --- | --- | --- | --- |
| 1 | 15.94 | 1344 | 0.11 | Triacetin |
| 2 | 18.17 | 1441 | 0.11 | Methyl oleate ozonide |
| 3 | 19.23 | 1460 | 0.71 | 2-Carboxymethyl-3-n-hexylmaleic acid anhydride |
| 4 | 20.07 | 1492 | 0.22 | 2-Allyl-5-t-butylhydroquinone |
| 5 | 20.28 | 1509 | 0.20 | Myristicine |
| 6 | 21.51 | 1514 | 0.19 | Pentadecanoic acid |
| 7 | 22.54 | 1560 | 0.12 | 1,7-Dimethyl-5-phenyltricyclo[4.1.0.0~2,7~]hept-3-ene |
| 8 | 24.34 | 1687 | 0.16 | 1-Tetradecanol |
| 9 | 24.89 | 1749 | 0.11 | (2-Phenyl-1,3-dioxolan-4-yl)methyl (9e)-9-octadecenoate |
| 10 | 25.01 | 1753 | 0.10 | Methyl tetradecanoate |
| 11 | 26.00 | 1788 | 0.55 | Tetradecanoic acid |
| 12 | 26.26 | 1796 | 1.26 | Cyclohexanone, 2-benzylidene- |
| 13 | 26.98 | 1807 | 0.76 | 1,2,3,6-tetramethylbicyclo[2.2.2]octa-2,5-diene |
| 14 | 27.74 | 1823 | 0.39 | 14-Pentadecenoic acid |
| 15 | 28.05 | 1834 | **3.63** | **8,12-Epoxy-13,14,15,16,17-pentanorlabdane** |
| 16 | 28.43 | 1840 | 0.23 | 11-Octadecenal |
| 17 | 28.70 | 1853 | 0.20 | Methyl 11-(3-pentyl-2-oxiranyl)undecanoate, cis- |
| 18 | 28.86 | 1858 | 0.38 | Actinomycin C2 |
| 19 | 28.94 | 1863 | 0.51 | Methyl 14-methylpentadecanoate |
| 20 | 29.19 | 1874 | **3.14** | **Methyl hexadecanoate** |
| 21 | 29.68 | 1894 | 0.97 | 2,3-Dihydroxypropyl palmitate |
| 22 | 30.56 | 1922 | **4.72** | **Hexadecanoic acid** |
| 23 | 30.90 | 1927 | 0.15 | 2-Hydroxy-3-(palmitoyloxy)propyl palmitate |
| 24 | 31.25 | 1944 | 2.62 | 5-Hydroxy-2,2-dimethyl-5,6-bis-(2-oxo-propyl)-cyclohexanone |
| 25 | 31.49 | 1986 | 0.99 | 14-Bromopentadecanoic acid |
| 26 | 31.51 | 1989 | 0.35 | 14-Hydroxy-15-methyl-15-hexadecenoic acid |
| 27 | 32.08 | 2003 | 1.74 | Methyl 11-(3-pentyl-2-oxiranyl)undecanoate, trans- |
| 28 | 32.35 | 2022 | **3.00** | **Linoleic acid, methyl ester** |
| 29 | 32.52 | 2034 | **4.50** | **Oleic acid, methyl ester** |
| 30 | 32.63 | 2076 | 1.13 | Elaidic acid, methyl ester |
| 31 | 33.01 | 2128 | 2.88 | Methyl stearate |
| 32 | 33.19 | 2143 | 0.14 | Arachidonic acid methyl ester |
| 33 | 33.84 | 2150 | **28.88** | **Oleic Acid** |
| 34 | 34.41 | 2172 | **7.89** | **Octadecanoic acid** |
| 35 | 34.57 | 2184 | 1.94 | Methyl-(5e,8e,11e,14e)-5,8,11,14-icosatetraenoate |
| 36 | 34.71 | 2199 | 0.33 | 9,10-Secocholesta-5,7,10(19)-triene-3á,25,26-triol |
| 37 | 34.83 | 2211 | 0.22 | 1-Heptatriacotanol |
| 38 | 35.11 | 2223 | 1.83 | 9,12-Octadecadienoic acid (Z,Z)- |
| 39 | 35.18 | 2229 | 1.63 | trans-9-Octadecenoic acid, pentyl ester |
| 40 | 35.29 | 2241 | 0.28 | Linoleic acid ethyl ester |
| 41 | 35.65 | 2259 | 1.29 | Stearic acid, allyl ester |
| 42 | 36.04 | 2277 | 0.35 | cis-11-Eicosenoic acid |
| 43 | 36.78 | 2322 | 0.09 | Oleic acid, eicosyl ester |
| 44 | 37.54 | 2383 | 0.14 | n-Butyl ricinoleate |
| 45 | 37.94 | 2396 | 0.62 | Linolein, 2-mono- |
| 46 | 38.05 | 2411 | 2.34 | 2,3-Dihydroxypropyl elaidate |
| 47 | 38.15 | 2423 | 2.02 | Olein, 2-mono- |
| 48 | 38.49 | 2444 | 1.03 | Stearic anhydride |
| 49 | 38.61 | 2456 | 0.73 | Isochiapin B |
| 50 | 38.82 | 2477 | 0.40 | Fumaric acid, hexadecyl 2-hexyl ester |
| 51 | 38.89 | 2481 | 0.10 | Docosanoic acid, 1,2,3-propanetriyl ester |
| 52 | 39.17 | 2490 | 0.96 | 3,3-Ethylenedioxy-5à-cholestane |
| 53 | 39.40 | 2498 | 0.96 | 1,2-Benzenedicarboxylic acid, bis(2-ethylhexyl) ester |
| 54 | 39.68 | 2508 | **4.15** | **Bis(6-methylheptyl) phthalate** |
| 55 | 40.83 | 2530 | 0.37 | Behenic acid methyl ester |
| 56 | 41.20 | 2569 | 1.10 | (Z,Z)-1,3-Dioctadecenoyl glycerol |
| 57 | 41.36 | 2612 | 0.49 | Ethyl iso-allocholate |
| 58 | 42.08 | 3023 | 0.87 | Methyl lignocerate |
| 59 | 45.48 | 3203 | 0.54 | Stigmast-5-en-3-ol |
| 60 | 47.17 | 4025 | 2.03 | Rhodopin |
| **Total** | | | **99.85** |  |
